# Women’s Experiences of Contraceptive Counseling for Informed Choice in Moshi Municipality, Tanzania: A Qualitative Study

**DOI:** 10.3390/healthcare14132020

**Published:** 2026-07-07

**Authors:** Angela Genes Lyimo, Kristin Akerjordet, Paulo Kidayi, Joseph Mlay, Eleanor L. Stevenson, Christina Furskog Risa

**Affiliations:** 1 Department of Midwifery, School of Nursing, KCMC University, Moshi P.O. Box 2240, Tanzania; 2 SHARE-Center for Resilience in Healthcare, Faculty of Health Sciences, University of Stavanger, P.O. Box 8600, N-4038 Stavanger, Norway; kristin.akerjordet@uis.no; 3 Faculty of the Arts, Social Sciences, and Humanities, School of Psychology, University of Wollongong, Wollongong, NSW 2522, Australia; 4 Department of Community Health, School of Nursing, KCMC University, Moshi P.O. Box 2240, Tanzania; paulo.kidayi@kcmcu.ac.tz; 5 Department of Obstetrics and Gynecology, School of Medicine, KCMC University, Moshi P.O. Box 2240, Tanzania; mlayjoseph507@gmail.com; 6 School of Nursing, Duke University, 307 Trent Drive, Durham, NC 27710, USA; eleanor.stevenson@duke.edu; 7 Department of Caring Ethics, Faculty of Health Science, University of Stavanger, P.O. Box 8600, N-4038 Stavanger, Norway; christina.f.risa@uis.no

**Keywords:** informed contraceptive choice, contraceptive counselling, provider–client interaction, women’s experiences, family planning services

## Abstract

**Background**: Tanzania’s national family planning standards mandate that healthcare providers deliver objective, comprehensive, and client-focused contraception information to enable informed choice. Despite these initiatives, evidence indicates contraceptive uptake in Tanzania is low (38%), with a growing rate of discontinuation at 34%. Therefore, this study aimed to explore women’s experiences of contraceptive counseling, with a particular focus on how information provided by healthcare providers influences informed contraceptive choice in Moshi Municipality, Tanzania. **Methods**: Semi-structured qualitative interviews were carried out with 15 women attending family planning clinics for contraceptive counseling at the two selected public health facilities in Moshi Municipality. Purposive sampling was used. Reflexive thematic analysis described by Braun and Clarke was employed. **Results**: Three main themes and eight sub-themes emerged. The main themes were information and communication gaps, provider interaction and autonomy, and social networks and structural influence. The sub-themes were insufficient and unclear information; myths and misconceptions; limited visual aids and practical demonstrations; respectful and friendly services; time given by the provider; provider-led choice, influence by peer stories and fear; and accessibility of the services and environment. Participants experienced limited discussion of available contraceptive methods and limited use of visual aids for thorough explanation to enable women to make an informed choice and consistent use of contraceptive methods. **Conclusions**: The findings highlight that women’s experiences with contraceptive counseling for informed contraceptive choice are diverse, including both positive and negative aspects. Respectful and friendly approaches and a supportive service environment are important; however, they are insufficient on their own to ensure informed contraceptive choice. Quality information from the healthcare provider that is clear, complete, accurate, and comprehensive is central to the counseling process. The integration of these factors is essential to empower women to make informed contraceptive choices that align with their reproductive intentions.

## 1. Introduction

Informed contraceptive choice is a cornerstone of quality family planning services and a fundamental component of a human rights-based approach to sexual and reproductive health [1]. According to the WHO, informed contraceptive choice is defined as when an individual makes an autonomous decision on the preferred contraceptive method after being given complete and clear information regarding all contraceptive method options, including how to use them, how effective they are, who can use and who cannot use them, expected side effects, and how to manage them [2]. Evidence shows women’s ability to make informed contraceptive decisions depends not only on access to services but also on the quality, completeness, and clarity of information provided during counseling encounters [3]. Informed choice during contraceptive counseling enables women to select contraceptive methods that best suit their reproductive goals and lifestyles, which influences contraceptive adoption and reduces contraceptive discontinuation [4].

Globally, clinical guidelines and policy frameworks highlight informed choice as a crucial metric for evaluating the quality of family planning services. Evidence indicates that there are still discrepancies between policy goals and routine counseling procedures in health facilities. Emerging evidence from low- and middle-income settings like Tanzania shows that women with poor and low health literacy levels are often made to adopt contraceptive methods without being fully involved in the decision process [5]. Contraceptive counseling is characterized by limited discussion of available options and provider suggestions [6]. Women’s contraceptive decision-making may also be influenced by social and structural factors that intersect with counseling encounters, such as partner influence, peer narratives, and health system pressures like time constraints and high client volumes [6]. Women’s autonomy in choosing a contraceptive method may be compromised, and false information may be spread if these influences are not addressed during counseling [7].

To facilitate informed decision-making, Tanzania’s national family planning guidelines emphasize that healthcare providers must give objective, comprehensive, and client-centered contraceptive information [8]. Despite the efforts, contraceptive uptake in Tanzania remains low at 38%, not achieving the national and global targets of 60% [9]. In addition, contraceptive discontinuation remains high at 34%, with frequent method switching reported at 4% [10]. A study conducted in the northern part of the Tanzania region with high contraceptive uptake indicates that 46% of women discontinue contraceptive methods while still in need [11]. Most of the existing research concentrates on contraceptive prevalence, method mix, and service consumption. Given the lower prevalence, there is a growing rate of discontinuation across the country. There is a need for context-specific exploration of women’s experiences of contraceptive counseling from their perspective.

In cases where counseling has been studied, evaluations usually depend on numerical measures of service quality, which may ignore the complex contextual, interpersonal, and informational elements that affect well-informed contraceptive choice [12]. Women’s experiences during provider–client contacts frequently influence not just their initial use of contraceptives but also their satisfaction, continuation, and faith in health services [13]. Therefore, the purpose of this study was to explore women’s experiences of contraceptive counseling, with a particular focus on how information provided by healthcare providers supports informed contraceptive choice in Moshi Municipality, Tanzania. By focusing on women’s perspectives in a local context, this study advances knowledge about the quality of counseling and offers data to support initiatives to improve client-centered and rights-based family planning services that ultimately improve contraceptive uptake and retention.

## 2. Materials and Methods

### 2.1. Study Design

This study adopted an exploratory, descriptive, qualitative design using a semi-structured interview guide to explore women’s experience of contraceptive counseling.

This design was deemed appropriate, as the study aimed to provide a systematic, context-specific description of contraceptive counseling encounters [14]. A semi-structured interview guide containing open-ended questions enabled participants to describe their experiences in their own words, facilitating in-depth exploration of how counseling processes influenced informed contraceptive choice (Appendix A).

### 2.2. Study Setting

The study was conducted in two high-volume public health facilities in Moshi Municipality, Kilimanjaro Region, Tanzania. The selected facilities were purposively chosen for their high client volume, which allows for multiple provider–client counseling interactions. Both facilities have reproductive and child health (RCH) units that offer daily family planning services. Each clinic provides family planning services to roughly 300–600 women per month. Eight trained nurse-midwives per facility rotate among prenatal care, labor and delivery, postnatal wards, and the family planning clinic. One nurse-midwife works in the family planning unit each day and offers a complete spectrum of contraceptive methods certified by the Tanzanian Ministry of Health, including short-acting, long-acting reversible, and permanent methods [8]. Trained nurse-midwives give contraceptive counseling in private rooms, and services are provided for free on a first-come, first-served basis during regular clinic hours.

### 2.3. Reflexivity

Data collection was conducted by the first author (AL), a female nurse-midwife with formal training and professional experience in sexual and reproductive health and contraceptive counseling. At the time of the study, AL was affiliated with KCMC University and had prior experience conducting qualitative research and in-depth interviews. The interviewer shared similar sociodemographic characteristics with participants, including gender and cultural background, which facilitated rapport and open communication. There was no prior relationship between the interviewer and the participants before recruiting. Before agreeing to participate, participants learned about the interviewer’s professional experience, institutional affiliation, and the study’s aim. As an academic midwife and researcher, the interviewer acknowledged that participants may have perceived her as an expert. I was aware that participants might view me as an expert; however, this was addressed through reflexive strategies, including team discussions and continuous critical reflection. We also considered potential power imbalances during interviews and minimized these by emphasizing the researcher’s role, fostering a nonjudgmental environment, and employing indirect questioning techniques to encourage honest answers. Therefore, everyday language was used, offered space for participants’ own priorities, and remained attentive to any signs of discomfort during the interview. The research team included specialists with backgrounds in midwifery, nursing, obstetrics and gynecology, and qualitative research. However, all researchers were conscious and actively acknowledged their own beliefs, biases, and judgment systems about family planning [15]. Throughout the study, the research team had reflective talks to identify and reduce potential biases based on their professional responsibilities and prior views about contraceptive services.

### 2.4. Participants and Recruitment

A purposive sampling strategy was used to recruit women upon arrival prior to receiving services. The interview was conducted immediately following the contraceptive counseling encounter; interview questions were based on the current visit. Ensuring that participants had direct experience relevant to the study aim, including women seeking to initiate contraceptive use and those wishing to resume contraception, for example, postpartum women, and those fluent in the Kiswahili language. The first author approached women upon arrival, and those who met the inclusion criteria were asked to participate in the study. Later on, they received both verbal and written information on the study, including its goal, methods, risks, and benefits. Those who consented to participate were asked to sign written informed consent. A total of 15 women were recruited at both research sites. No eligible participants declined to participate or withdrew after providing consent. Recruitment continued until sufficient information power was achieved, guided by ongoing reflection on the depth and richness of the data [16]. Although data saturation was observed after 13 participants, two additional interviews were conducted to confirm that no new themes emerged [17].

### 2.5. Data Collection

Data were collected in December 2024 using a semi-structured interview guide developed based on the Tanzanian National Family Planning Guidelines, with a specific focus on elements related to informed contraceptive choice [8]. The interview guide included open-ended questions and probing prompts to explore women’s experiences of contraceptive counseling, information provision, decision-making, and interaction with healthcare providers (Appendix A). The interview guide was piloted with three women at a separate health facility with similar characteristics to the study area, and minor refinements such as rewordings of unclear questions and reordering questions were made to improve clarity and flow. The data from the pilot was not included in this study.

Face-to-face interviews were conducted in a quiet, private area outside the health care facility to the nearby prepared area to maintain confidentiality and promote open conversation. The first author conducted all interviews in Kiswahili, and, with consent from the participants, the conversation was audio recorded to ensure all information was captured. Every interview lasted between thirty to sixty minutes. Contextual observations and preliminary analytical thoughts were recorded by taking field notes during and immediately after interviews. Data collection and preliminary analysis occurred concurrently. Recruitment was concluded when no new themes or substantive insights emerged from successive interviews, indicating adequate data saturation and information power for the study objectives.

### 2.6. Data Analysis

Audio-recorded interviews were transcribed verbatim in Kiswahili by the first author and subsequently translated into English for analysis. Backward translation was used to preserve the original meaning of cultural concepts of the Swahili language by an independent translator. To safeguard participant identity, transcripts were made anonymous. Data were analyzed using the reflexive thematic analysis approach described by Braun and Clarke, which included the following six phases: (1) becoming familiar with the data, (2) generating initial codes, (3) searching for themes, (4) reviewing themes, (5) defining and naming themes, and (6) producing the report [18]. This approach allowed patterns of meaning to emerge inductively from participants’ accounts while supporting systematic interpretation. The first author’s and the last author’s re-read the transcripts to grasp the overall concept of the content. The data material was shared through a protected data storage backup (external hard disk). The transcripts were coded independently by the first and last authors, and then co-coding was done by the same authors. Finally, online discussions were held to ensure all codes were performed similarly. The theme and subthemes emerged from the participants’ information after reaching consensus on the discussion between all authors. The original meaning of the data was retained throughout the data analysis. This is a process of rendering participants’ original information. The authors finally reached a consensus on three main themes with a total of eight sub-themes. Qualitative data analysis was conducted manually rather than using software, as the small sample size allowed close engagement with the data.

### 2.7. Trustworthiness

Consideration was given to credibility, dependability, confirmability, and transferability to establish trustworthiness as per Lincoln and Guba [19]. Long-term interaction with the data, verbatim transcription, and the use of participant quotes to support findings all increased credibility. The interview guide was validated and pretested to ensure its reliability, and one researcher conducted the interview using the same tool, which ensured the quality of information gathered, thus enhancing dependability. To reduce researcher bias, the study team’s reflexive talks bolstered confirmability. Giving thorough explanations of the study’s background, participants, and methods helped to promote transferability.

## 3. Results

### 3.1. Participant Characteristics

A total of 15 women who attended a family planning clinic for contraceptive counseling were engaged in the study. The mean age was 27 years (SD = 5.7), with the youngest being 18 and the oldest 48 years. Furthermore, 20% (n = 3) of the participants were single (Table 1).

### 3.2. Themes and Sub-Themes

**Figure 1:**
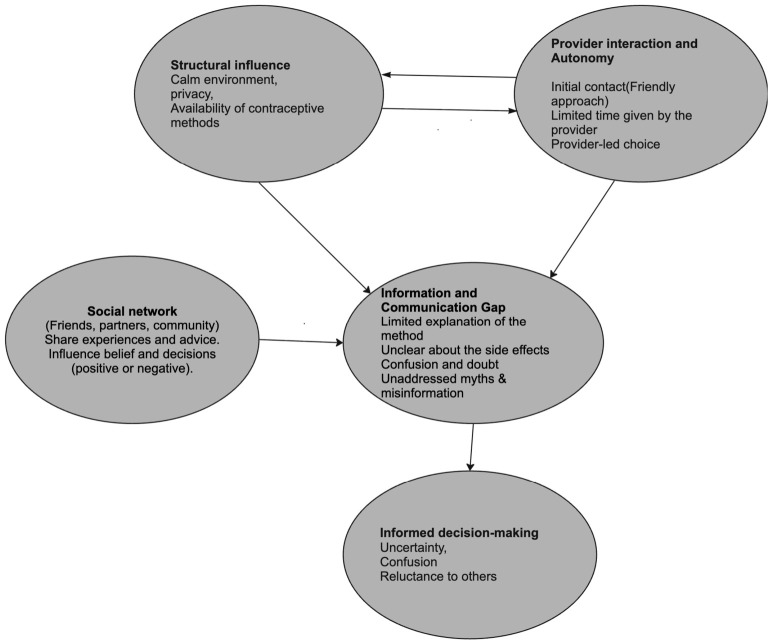
Thematic map.

Three main themes and eight sub-themes emerged from the analysis: (i) Information and communication gap, (ii) Provider interaction and autonomy, and (iii) Social and structural influence. The main themes and sub-themes are presented in Table 2 and its thematic map, which shows the relationship between themes and sub-themes and how they influence informed contraceptive choice in Figure 1.

### 3.3. Theme 1: Information and Communication Gap

Participants’ narratives reflected experiences of insufficient, unclear contraceptive information and were narrowly focused on a single preferred contraceptive method, which limited their ability to make an informed contraceptive choice. Several participants indicated that the information provided was incomplete, myths went unaddressed, and visual aids and demonstrations were absent, all of which are essential components for supporting informed contraceptive choice. Interview accounts suggested that inadequate counseling information limited women’s ability to evaluate available contraceptive options, as they lacked the comprehensive understanding needed to evaluate available options and align decisions with their reproductive intentions.

#### 3.3.1. Insufficient and Unclear Information

Accounts revealed that counseling sessions were often narrowly focused when they discussed only the preferred contraception method that was asked prior to contraceptive counseling, limiting opportunities for informed contraceptive choice. Instead of providing a thorough review of potential options available, counseling frequently focused solely on the aforementioned contraceptive method, which limited opportunities to compare alternative contraceptive methods and consider other possibilities. This approach left several women feeling that their awareness of the full spectrum of contraceptive options was limited and that their decisions were made without the comprehensive information necessary for an informed contraceptive choice.

*“I wished that she could explain all methods …because I might have made a different decision.”*

[Facility M, p. 6]

*“She just asked what contraceptive method I prefer…I mentioned implant and then started to explain about implants… She didn’t explain about the other method.”*

[Facility P, p. 9]

Moreover, some women frequently described receiving vague or partial explanations about contraceptive methods, which undermined their ability to make an informed contraceptive choice. Critical details such as how methods work, duration of protection, onset of effectiveness, and management of side effects were often omitted or only briefly mentioned, limiting the information necessary for an informed contraceptive choice. Several women also felt that different contraceptive methods were presented as having similar side effects, further constraining their ability to weigh options and make an informed contraceptive choice.

*“The nurse-midwife said that all contraceptive methods have similar side effects, whether pills, implants, injections, or IUDs. She did not explain what side effects belong to which method.”*

[Facility M, p. 10]

Some women’s narratives also revealed ambiguity about essential method-related instructions, especially where explicit assistance was expected, which hindered their ability to make an informed contraceptive choice. Inconsistent instructions left several women unclear about what to do after receiving a method and how to evaluate information from various sources, further constraining their capacity for an informed contraceptive choice.

*“She didn’t tell me when to have sex without a condom after the implant. My friend was told to have sex after three days, but I used to know it was safe after seven days, so I’m not sure if things have changed.”*

[Facility P, p. 12]

Furthermore, information about follow-up and continuity of care was inconsistent and not routinely provided, limiting women’s ability to sustain an informed contraceptive choice. Most women were advised to return only if problems occurred, with no clear guidance on routine follow-up, which is essential for maintaining an informed contraceptive choice over time.

*“She did not tell me when to come back. She only said I should return if I had a problem.”*

[Facility M, p. 9]

Other women reported receiving few instructions immediately following the administration of the contraceptive method, with little information about what would happen next or what to expect in the coming months. In these circumstances, follow-up information seemed to be provided only when women themselves asked for clarification.

*“She injected me, and then… she said I can go home… I just asked when to come back; the nurse responded, ‘After three months, I have to come to be injected again.’”*

[Facility M, p. 5]

#### 3.3.2. Myths and Misconceptions

Participants indicated myths and rumors about contraceptive methods, particularly intrauterine devices, were not regularly addressed or clarified during counseling sessions. Accounts suggested that issues rooted in community narratives were occasionally highlighted during consultations but were not always addressed in a way that reassured or enlightened them. As a result, these worries continued to influence perceptions and contraceptive choices.

*“Some people say the intrauterine device (IUD) can disappear in the body or even kill you. I asked the nurse if that was true, but she did not respond to that question and just continued talking about something else.”*

[Facility M, p. 2]

In other cases, some women recalled deciding to avoid specific procedures because of anxieties they had created before counseling, with no conversation to address or clarify these concerns during counseling, thereby leaving these concerns unresolved. When women voiced uncertainty, counseling occasionally switched toward picking an alternative strategy rather than addressing the underlying beliefs:

*“I told the nurse I don’t want an IUD (intrauterine device), as they cause cancer in the future… She asked me to choose another contraceptive method, and I decided to use an implant.”*

[Facility P, p. 7]

#### 3.3.3. Limited Visual Aids or Practical Demonstration of Contraceptive Methods

Interview data demonstrated that healthcare providers relied predominantly on verbal explanations, making it difficult for clients to understand the concepts and identify the contraceptive methods they wanted. Some women indicated that this reliance on verbal communication during counseling limited their ability to clearly differentiate between options. They expressed that seeing or handling contraceptive methods would have provided them with a deeper understanding of their choices, empowering them to make informed contraceptive decisions. The absence of demonstrations, models, or educational materials diminished their comprehension and confidence in selecting the most suitable methods. Women emphasized the necessity for healthcare providers to use visual aids to enhance their understanding and facilitate informed contraceptive choices, rather than depending solely on verbal information during interactions.

*“She mentioned the implant, but she did not show it to me. I don’t know how it looks or feels… I wish she had shown me during the counseling so that I could be sure about my choice before deciding.”*

[Facility P, p. 6]

In absence of visual explanations, some women admitted to relying on approaches they were already familiar with rather than exploring new possibilities. Fear of the unknown, together with confusion about how prior approaches arose or were used, influenced their willingness to attempt something new:

*“I choose injection because I was using it before… She just mentioned other methods, but I don’t know what they look like or where they will be placed. I was afraid of taking something new”.*

[Facility M, p. 5]

### 3.4. Theme 2: Provider Interaction and Autonomy

This theme captures how relational dynamics between the provider and the woman shaped the counseling encounter and influenced women’s comfort, engagement, and participation in decision-making. In this section, the findings related to the provider interaction and autonomy include a respectful and friendly approach, women’s hesitation to engage due to perceived limited time given by the provider, and provider-led contraceptive choice.

#### 3.4.1. Respectful and Friendly Approach

Some of the participants expressed that the providers were largely polite and respectful and used welcoming language. Some expressed that the provider had given them the opportunity to ask questions. This appeared to foster comfort, openness and trust during counseling interactions, as expressed by the following participant. This also strengthened trust between women and providers when weighing the best option for a contraceptive method during contraceptive counseling:

*“The nurse welcomed me warmly…and asked if I wanted anything…. I responded, ‘No, only contraceptive counseling for today’”.*

[Facility P, p. 11]

*“The midwife was charming and friendly; I was very comfortable and relaxed”.*

[Facility P, p. 5]

Furthermore, some women described after initial greetings, particularly through efforts to inquire about a woman’s personal well-being before initiating contraceptive counseling.

Participants’ accounts revealed some providers were taking time to ask about their general health, family situation, or overall well-being. This approach signals genuine care beyond the clinical task, helping women feel acknowledged as individuals rather than just recipients of a service.

*“The nurse first asked me how I was doing and how my family is before we started talking about family planning. That made me feel cared for.”*

[Facility M, p. 6]

#### 3.4.2. Time Given by the Providers

Participants described counseling interactions as brief and constrained by workload pressures. According to women’s testimonies, the pace of counseling influenced both the depth and quality of interaction, leaving little room for personalized discussion, explanation, or reflection. The perceived urgency of the interaction did not influence women’s readiness to ask questions and raise concerns, as some were aware of other clients waiting to be seen. Some women interpreted the opportunity to ask questions as symbolic rather than practical, particularly when counseling sessions were perceived to be rushed, which may negatively affect women’s participation in contraceptive decision-making.

*“There were many people waiting, so she seemed in a hurry. I felt there was not enough time to ask everything I wanted.”*

[Facility M, p. 8]

*“The time was not enough…many people were waiting…she appeared to be in a hurry.”*

[Facility M, p. 13]

#### 3.4.3. Provider-Led Contraceptive Choice

Although women were frequently questioned about their choices, some believed that providers made decisions based more on their own assessments than on what the women wanted. In certain instances, women accepted provider advice without being given any justifications:

*“I wanted the five-year implant, but she told me to use pills first. She did not explain why, and I did not question her.”*

[Facility M, p. 2]

On the other hand, women reported feeling free to express their preferences regarding contraceptive methods; however, they also encountered provider bias. This bias manifested itself through explicit or implicit recommendations for specific methods and the discouragement of others. Often, personal prejudices or assumptions about what would be suitable for the client influenced the providers’ judgments, overshadowing their autonomy.

*“I wanted an implant for five years because I don’t want another baby soon, but the nurse told me I had to use pills for a few months before I could get the implant. I didn’t ask for an explanation, and the provider didn’t clarify why she prescribed pills instead of the implant I wanted. She just gave me the pills for three months.”*

[Facility M, p. 2]

*“She just tells me to use injections… but I wanted pills”.*

[Facility M, p. 10]

### 3.5. Theme 3: Social Network and Structural Influence

Women’s contraceptive choices were shaped not only by the counseling provider but also by peers and myths. When clinical counseling did not directly counter misinformation, informal networks often prevailed.

#### 3.5.1. Being Influenced by Peer Stories and Fears

Most of the women described that their contraceptive choices for contraceptive methods were influenced to some degree by advice and stories from peers. This was further complicated by community myths, which included both accurate and inaccurate information perpetuated by peers and friends, ultimately impacting their informed contraceptive choices:

*“My friend told me to use implants…she said other methods can cause cancer…she advised against [IUD]. Therefore, I decided to follow my friend’s suggestion”.*

[Facility M, p. 7]

*“I didn’t choose an IUD, as I heard it can cause infertility… I have only one child, so I don’t want to risk”.*

[Facility P, p. 3]

#### 3.5.2. Accessibility of the Services and Environment

Participants described the service as available and free of charge and were positive about the environment where contraceptive counseling was conducted, especially the cleanliness of the room, calmness, and privacy. Women narrated the presence of free services and availability of all contraceptive methods as a motivation to choose any method they wanted without being limited to either cost or its availability hence it facilitated their decision for contraceptive preferences.

*“The counseling room was calm and private so that no one could hear what we were talking about with the provider.”*

[Facility M, p. 14]

*“I didn’t ask to pay anything; the service was free of charge.”*

[Facility P, p. 5]

## 4. Discussion

This study in Moshi Municipality, Tanzania, explored women’s experiences with contraceptive counseling for informed contraceptive choice. The study revealed that contraceptive making was shaped by multiple interacting influences, such as access to quality information, supportive healthcare interactions, structural conditions, and social network influences. Effective counseling, which includes myth-responsive communication, visual aids, provider time, and follow-up, enables informed decisions. In contrast, a lack of these factors may lead to uncertainty and reliance on familiar contraceptive methods.

This study found a significant lack of quality and comprehensive information in contraceptive counseling, with discussions often focusing on one “preferred” method rather than offering balanced comparisons. This contradicts WHO and Tanzanian guidelines, which call for complete, client-centered information to support informed choice. Inadequate counseling is linked to dissatisfaction and limits women’s ability to make informed decisions. Similarly, studies in Sierra Leone found 37% of clients received incomplete information and 17% received none [20]. Women’s ability to compare options was limited by insufficient method-specific explanations and generalized side effects, a trend also seen in other low- and middle-income countries, including Zambia, where side effects were under-explained [21]. This deficiency may reflect time constraints, emphasis on contraceptive uptake, and limited prioritization of individualized counseling.

An additional issue identified in our study is the lack of visual aids and an excessive dependence on verbal explanations during contraceptive counseling. The lack of visual aids exacerbates misunderstandings; these tools are advised to organize discussions, clarify method comparisons, and facilitate decision-making [22]. In contexts with diverse literacy levels and generally low health literacy, like the setting of this study, a one-dimensional approach limits women’s ability to fully understand and engage with their contraceptive choices [23]. These findings align with other studies that highlight how the lack of audiovisual and interactive decision-making tools significantly limits the counseling experience, especially when discussing complex options such as implants or IUDs [24]. These findings suggest the need to strengthen contraceptive counseling approaches by incorporating more interactive and client-centered education strategies. Providing clients with relevant, accessible, and diverse educational resources can transform counseling from passive information delivery into active engagement, helping women make decisions that truly reflect their values, circumstances, and long-term reproductive goals [25]. The use of tiered charts to communicate contraceptive effectiveness has proven to increase method-specific knowledge and improve informed contraceptive choice for long-acting reversible contraceptives [26].

The study also showed that during counseling sessions, myths and prejudices that were prevalent in communities were not always addressed. According to women’s testimonies, unresolved issues with implants, intrauterine devices, and perceived health hazards remained following counseling, which increased rather than decreased doubt. This result is consistent with research from sub-Saharan Africa that demonstrates how unchecked rumors and peer narratives can erode confidence in contraception and lead to the avoidance or abandonment of specific methods [27]. Our findings correlate with a study done in South Africa, which reported that perceived barriers such as infertility, changes in vaginal wetness, various cancers, and changes in body image contributed to poor contraceptive uptake among adolescent and young women [27].

Particular contraceptive methods were to some extent influenced by advice and stories from friends, spouses, and community members. Social networks often serve as informal yet powerful sources of reproductive health knowledge, especially in areas where formal education or open discussion is limited [28]. Peer experiences, whether positive or negative, tend to shape perceptions of safety, efficacy, and acceptability of various methods [28]. For some individuals, the endorsement or criticism of a contraceptive method by a trusted friend carried more weight than clinical advice, especially when there was mistrust in the healthcare system or unclear information from providers [29]. In our study, providers who began conversations respectfully and warmly made women feel comfortable during contraceptive counseling. Women who did not experience disrespect or abuse were significantly more likely to make informed contraceptive choices, highlighting the importance of a respectful, welcoming approach for open dialogue [30]. In this study, while clients felt emotionally supported, they lacked sufficient technical information to make informed choices. Friendly counseling alone left them uninformed about key contraceptive details, limiting its effectiveness. The study underscores the need to balance empathy with accurate information for truly informed contraceptive choice.

### Strength and Limitations

This study uses a qualitative approach to deeply explore women’s experiences with contraceptive counseling, highlighting real-life factors that influence informed contraceptive choices and providing insights often missed by quantitative research. However, it is equally important to recognize its limitations. The study was carried out at only two high-volume sites; nevertheless, transferability should be interpreted cautiously and may apply primarily to similar urban healthcare settings. Additionally, the data were collected from women’s self-reported experiences, which may have been influenced by social desirability.

## 5. Conclusions

The findings highlight that women’s experiences with contraceptive counseling for informed choice are diverse, including both positive and negative aspects. Respectful and friendly approaches and a supportive service environment are important; however, they are insufficient on their own to ensure informed decision-making. Quality information from the healthcare provider that is clear, complete, accurate, and comprehensive is central to the counseling process. Integration of these elements may strengthen women’s ability to make well-informed contraceptive decisions aligned with their reproductive intentions.

Contraceptive service stakeholders (healthcare professionals, policymakers, and service planners) should aim at empowering healthcare providers through training on contraceptive counseling and development of policy related to women’s and healthcare providers’ empowerment during contraceptive counseling interactions for informed decision-making. Future interventional studies are recommended to address the healthcare providers’ perspectives to understand the underlying factors that hinder the quality of information provided to the clients during contraceptive counseling interactions.

## Figures and Tables

**Table 1:** Socio-demographic characteristics of the study participants (n = 15).

Variables	Frequency (n)	Percent (%)
Age group (years)		
- 15–24	3	20
- 25–33	9	60
- >33	3	20
Education		
- Primary	5	33
- Secondary	9	60
- College or University education	1	7
Occupation		
- Employed	3	20
- Unemployed	12	80
Marital status		
- Single	3	20
- Cohabiting	6	40
- Married	6	40
Parity		
- Nullipara	1	6
- Parous	4	27
- Multipara	10	67

**Table 2:** A thematic overview showing themes, subthemes, and codes relating to women’s experience of contraceptive counseling.

Theme	Subtheme	Codes
Information and Communication Gap	Insufficient and Unclear Information	Unclear duration of method efficacy Unspecified onset of effectiveness Vague or generic side effect explanation Incomplete explanation of how methods work No explanation about managing side effects
	Myths and misconceptions	Myths not proactively clarified Persistent beliefs about implant, IUDs, and pills Missed opportunity to correct misconceptions
	Limited visual aid and practical demonstration	Not allowed to touch devices. No models or flyers provided Saw devices, but no hands-on demonstration Lack of visual aids for method explanation
Provider interaction and Autonomy	Friendly approach	Polite greeting Respectful language Warm greeting
	Limited time given by the provider	Time constraints The provider seemed rushed due to workload
Social network and structural influence	Influence by peer stories	Method choice influenced by friends/peers Community myths influence perceptions Lack of family/partner support as barrier
	Accessibility and Environment	Positive comments about environment/cost Heard about services from other women Calm place Availability of all contraceptive methods
	Provider-led contraceptive choice	The provider suggests the method without explanation Perceived provider expertise Client defers to the provider’s authority

## Data Availability

The data used and/or analyzed during the current study is available from the corresponding author on reasonable request to protect participant privacy and to comply with ethical requirements.

## Appendix A. Semi-Structure Interview Guide

**Discussion Topic**	**Core Question**	**Probes**
1. Background & Initial Contact	What brought you to the facility for contraceptive counselling today?	- Can you tell me about the person or situation that influenced your decision to come today? - What did you expect during your counselling session?
2. Information & Communication	How would you describe the information you received about contraceptive methods during counselling?	- How does the provider start the session. - Briefly explain about others methods you discussed. - What did the provider explain about how the method works and how long it lasts? - How did the provider respond when you mentioned any myths or rumors you had heard?
3. Provider Interaction & Autonomy	How did the provider involve you in deciding which method to use?	- Can you describe a moment when you felt able to ask questions freely during the session? - How did the provider explain their reasons for suggesting or not suggesting a method?
4. Social & Structural Influences	Outside the facility, who or what influences your contraceptive choices?	- Can you share a story you’ve heard in the community about a specific method? - How have the cost, waiting time, or facility environment shaped your decisions about contraception?
5. Follow-Up & Continuity	What guidance did you receive about follow-up after starting your method?	- What did the provider say about when or why you should return to the facility? - How would you like the follow-up process to be arranged so it works best for you?
